# miR-15a/16 reduces retinal leukostasis through decreased pro-inflammatory signaling

**DOI:** 10.1186/s12974-016-0771-8

**Published:** 2016-12-08

**Authors:** Eun-Ah Ye, Li Liu, Youde Jiang, Jenny Jan, Subhash Gaddipati, Susmit Suvas, Jena J. Steinle

**Affiliations:** 1 Department of Anatomy and Cell Biology, Wayne State University, 9314 Scott Hall, Detroit, MI 48202 USA; 2 Ophthalmology, Wayne State University, 9314 Scott Hall, Detroit, MI 48202 USA; 3 Immunology and Microbiology, Wayne State University, Detroit, MI USA

**Keywords:** miR-15a/16, IL-1β, TNFα, NF-κB, Leukostasis

## Abstract

**Background:**

Hyperglycemia is a significant risk factor for diabetic retinopathy and induces increased inflammatory responses and retinal leukostasis, as well as vascular damage. Although there is an increasing amount of evidence that miRNA may be involved in the regulation in the pathology of diabetic retinopathy, the mechanisms by which miRNA mediate cellular responses to control onset and progression of diabetic retinopathy are still unclear. The purpose of our study was to investigate the hypothesis that miR-15a/16 inhibit pro-inflammatory signaling to reduce retinal leukostasis.

**Methods:**

We generated conditional knockout mice in which miR-15a/16 are eliminated in vascular endothelial cells. For the in vitro work, human retinal endothelial cells (REC) were cultured in normal (5 mM) glucose or transferred to high glucose medium (25 mM) for 3 days. Transfection was performed on REC in high glucose with miRNA mimic (hsa-miR-15a-5p, hsa-miR-16-5p). Statistical analyses were done using unpaired Student *t* test with two-tailed *p* value. *p <* 0.05 was considered significant. Data are presented as mean ± SEM.

**Results:**

We demonstrated that high glucose conditions decreased expression of miR-15a/16 in cultured REC. Overexpression of miR-15a/16 with the mimic significantly decreased pro-inflammatory signaling of IL-1β, TNFα, and NF-κB in REC. In vivo data demonstrated that the loss of miR-15a/16 in vascular cells led to increased retinal leukostasis and CD45 levels, together with upregulated levels of IL-1β, TNFα, and NF-κB.

**Conclusions:**

The data indicate that miR-15a/16 play significant roles in reducing retinal leukostasis, potentially through inhibition of inflammatory cellular signaling. Therefore, we suggest that miR-15a/16 offer a novel potential target for the inhibition of inflammatory mediators in diabetic retinopathy.

**Electronic supplementary material:**

The online version of this article (doi:10.1186/s12974-016-0771-8) contains supplementary material, which is available to authorized users.

## Background

Hyperglycemia and diabetes have clear influences on the dysfunction of vascular endothelial cells, leukocyte adhesion, and inflammatory signaling [1–3]. However, the molecular and physiological mechanisms of diabetes-induced retinal damage are still not clear. Over the last decade, the role of microRNA (miRNA) as a mediator of pathological mechanisms in diabetic retinopathy has come to light. miRNA (miR) are small non-coding RNA molecules and post-transcriptional regulators, leading to reduced expression of target mRNAs. Based on current findings, the human genome encodes at least 1000 microRNAs [4] and approximately 30 to 60% of human genes are estimated to be regulated by miRNAs [5, 6].

miR-15a may be a key miRNA in the diabetic retina, as lower levels of miR-15a were found in the plasma of patients with prevalent diabetes mellitus (DM) [7]. In addition, miR-15a levels were decreased in high glucose conditions in human umbilical vein endothelial cells (HUVEC) [8]. Our previous study showed altered expression of miR-15 family members, miR-15b and miR-16, in retinal endothelial cells (REC) cultured in high glucose conditions [9]. In contrast, elevated levels of hsa-miR-15a were found in eyes with proliferative diabetic retinopathy (PDR) [10]. Also, it was reported that miR-15a was highly expressed in early endothelial progenitor cells [11].

REC are substantially affected in diabetic retinopathy. Our previous studies have shown regulatory effects of miRNA on insulin resistance [9] and inflammatory signaling [12] in human REC cultured under high glucose conditions. The pathology of diabetic retinopathy involves endothelial dysfunction, as well as activation of inflammatory signaling and leukocyte adhesion. Retinal leukostasis, a histological indication of retinal inflammation, can be induced by vascular endothelial growth factor (VEGF) [13, 14], which is a direct target of miR-15a in HUVEC [15]. In addition to retinal leukostasis, work has shown that diabetic retinas have elevated levels of inflammatory cytokines, such as TNFα and IL-1β [16]. Both TNFα and IL-1β levels can be regulated through toll-like receptor (TLR) signaling, which is reduced by miR-15a/16 in LPS-treated macrophages [17]. In addition to VEGF, pro-inflammatory signaling molecules (TLR5/8, IRAK1, TRAF6) are predicted targets of miR-15a (targetscan.org). NF-κB activation through TLR5 [18, 19] and TLR8 [20] has been shown in HEK 293 cells.

Thus, we aimed to investigate the hypothesis that miR-15a/16 inhibit the pro-inflammatory signaling of TNFα, IL-1β, and NF-κB to reduce retinal leukostasis. To perform this study, we utilized REC in normal and high glucose conditions for in vitro work and miR-15a/16-conditional knockout mice for in vivo analysis.

## Methods

### Cell culture

Human REC were acquired from Cell Systems Corporation (CSC, Kirkland, WA). Cells were grown in M131 medium containing microvascular growth supplement (Invitrogen), 10 μg/ml gentamycin, and 0.25 μg/ml amphotericin B. For experiments, cells were maintained in normal (5 mM) glucose or transferred to high glucose medium (25 mM) (Cell Systems) for 3 days. Only primary cells within passage 5 were used. Cells were quiesced by incubating in high or normal glucose medium without growth supplementation for 20 h and used to perform the experiments.

### Cell transfection with microRNA-mimics

REC were transfected with miRNA mimic (hsa-miR-15a-5p and hsa-miR-16-5p) (Invitrogen, Carlsbad, CA) using Oligofectamine (Invitrogen) following manufacturer instructions. miR-transfection was performed 48 h before cell harvest. A final concentration of 30 nM was used when transfected separately (miR-15a and miR-16), and 15 nM was used in combination (miR-15a + miR-16). Additionally, a 30-nM Mimic Negative Control (Invitrogen) was transfected into REC cultured in high glucose as a control. Other control groups, normal glucose (NG) and high glucose (HG), were treated with Oligofectamine only. The levels of miRNA overexpression were verified using quantitative reverse transcription polymerase chain reaction and real-time PCR.

### Real-time quantitative PCR

Total RNA was isolated and purified using the TRIzol method, and the purity and quantity of RNA were measured using Synergy HTX multi-mode reader (BioTek; Winooski, VT). For polyA tailing reverse-transcriptase PCR, 5 μg of total RNA was treated with DNase I for 15 min at room temperature (Promega; Madison, WI) and then added with polyA using (polyA) polymerase (NEB; Ipswich, MA) at 37 °C for 1 h. The final reaction mixtures were extracted with phenol/chloroform, precipitated with isopropanol, and redissolved in 25 μl diethylpyrocarbonate (DEPC)-treated water. PolyA-tailed RNA (6 μl) was reverse-transcribed into first-strand cDNA using SuperScript II reverse transcriptase (Invitrogen) with the oligo-dT adapter primer 5′GCGAGCACAGAATTAATACGACTCACTATAGGTTTTTTTTTTTTVN3′. For PCR, 1 μl of RT product was diluted three times and used as a template in each reaction. The reverse primer was from the adapter sequence 5′GCGAGCACAGAATTAATACGACTCAC3′ and the forward primers were specific to miR-15a and miR-16 mature sequences. U6 small non-coding RNA sequence was amplified as the internal control using the primers 5′GCTTGCTTCGGCAGCACATATAC (forward) and 5′TGCATGTCATCCTTGCTCAGGG3′ (reverse). The SYBR-Green-based real-time PCR was performed using the CFX Connect PCR system (BioRad; Hercules, CA). The relative expression of miRNA was calculated based on the formula 2^(−delta delta Ct)^. Delta-delta Ct values are delta Ct _exp._ − delta Ct _cont._.

### Generation of miR-15a/16 Cre-LoxP mice

miR-15a/16 floxed mice (B6-129S-mirc30.tm1.1rdf/J) and B6.FVB-Tg (cdh5-cre)7Mlia/J Cre mice were purchased from Jackson Laboratories. The miR-15a/16 floxed mice were crossed with cdh5-Cre mice to generate conditional knockout mice in which miR-15a/16 is eliminated in vascular endothelial cells. At 3 months of age, both male and female mice from miR-15a/16 floxed and miR-15a/16 Cre-LoxP groups were used for further analyses. All animal procedures were reviewed and approved by the Institute Animal Care and Use Committees of the Wayne State University School of Medicine (Protocol # A 11-08-14) and conform to NIH guidelines.

### Genotyping analysis

Genomic DNA from the ear punch of 2-week-old mice was digested with one-step tail DNA extract buffer (100 mM Tris, 5 mM EDTA, 200 mM NaCl, 1% Triton) plus proteinase K (10 mg/ml) at 55 °C overnight and then heat-inactivated at 85 °C for 45 min. Sequences of primer pairs used to screen the transgenic mice were as follows: (1) miR-15a/16- 5′→3′ forward: TCAGTTAACCAATAAAAA GGTCAGC, reverse: GCCTGGGTCTCACCA TGTAG; (2) Cdh5-cre: forward: AGGCAGCTCACAAAGGAACAAT, reverse: TCGTTGCATCGACCGGTAA; (3) Cdh5-cre internal positive forward: CTAGGCCACAGAATTGAAAGATCT, reverse: GTAGGTGGAAATTCTAGCATCATCC. Standard PCR reaction protocol was completed using KAPA2G HotStart Genotyping PCR Mix (KK5621, KAPA Biosystems). PCR reaction was performed with the following steps: denature 95 °C 3 min, 35 cycles at 95 °C, 15 s, 60 °C 15 s, 72 °C s/kb, and then final extension at 72 °C 1 min.

### Western blot analysis

After rinsing with cold PBS, REC or whole retinal lysates were collected in lysis buffer containing protease and phosphatase inhibitors. Equal amounts of protein were separated on precast Tris-glycine gels (Invitrogen, Carlsbad, CA) and then blotted onto a nitrocellulose membrane. After blocking in TBST (10 mM Tris-HCl buffer, pH 8.0, 150 mM NaCl, 0.1% Tween 20) and 5% (*w*/*v*) BSA, the membrane was treated with appropriate primary antibodies followed by incubation with secondary antibodies labeled with horseradish peroxidase. Antigen-antibody complexes were detected by chemilluminescence reagent kit (Thermo Scientific, Pittsburgh, PA). Primary antibodies used were phosphorylated NF-κB p65 (Ser 536) (Cat.# 3033), NF-κB p65 (Cat.# 4764) (rabbit monoclonal, 1:500; all purchased from Cell Signaling, Danvers, MA), and beta actin (Santa Cruz, Santa Cruz, CA). Imaging was performed using C500 system (Azure Biosystems, Dublin, CA) within a linear range of exposure. Signal intensity of protein bands was normalized by beta actin. Even staining of beta actin was observed after loading of equal amounts of protein.

### ELISA analysis

TNFα protein concentrations were measured using a TNFα ELISA (Thermo Fisher scientific, Pittsburgh, PA). Into all wells, 100 μg protein was loaded, with analyses based on a standard curve. A human IL-1β ELISA (R and D system, Minneapolis, MN) was used to measure levels of the pro-inflammatory cytokine, IL-1β, in cell lysates. Equal protein was loaded (100 μg) into all wells to allow for comparisons based on OD. Manufacturer’s instructions were followed for all ELISA, except extended incubation of samples and antibody reagents overnight at 4 °C.

### Leukostasis analysis

To assess leukostasis in the retina, we collected retinas from five mice each of the Cre-loxP and floxed only animals. Eye globes were enucleated and placed into 4% paraformaldehyde for 1 day. After rinsing in PBS, the retinas were dissected out gently and immunohistochemistry was performed. Briefly, retinas were incubated in 5% BSA for 2 h, followed by incubation in a cocktail of primary antibodies CD45 (Cat#. 550586, mouse monoclonal, 1:100, BD Pharmingen) and isolectin GS-IB4 conjugated with Alexa Fluo 488 (Cat#. I21411, 1:100, Life technologies) for 2 days at 4 °C. After rinsing in PBS, the retinas were incubated with goat anti-mouse Alexa Fluor 555 antibody (Cat# A21424, 1:500, Life technologies) overnight at 4 °C. The retinas were rinsed in PBS and flat mounted onto glass slides. Imaging analysis was performed using confocal microscopy (Leica, Buffalo Grove, IL).

### Flow cytometry analysis

Single-cell suspensions were prepared from neuronal retinal tissue of the miR-15a/16 floxed, miR-15a/16 cre/loxP mice by incubating with 0.05% trypsin for 30 min at 37 °C. The cell suspension was then passed through a 70-μm nylon mesh (Fisher Scientific). Fc (CD16/32) receptors were blocked with anti-CD16/32 (clone 2.4G2) antibody prior to cell surface staining. To stain cell surface antigens, all retinal cells were incubated in 100 μl of staining buffer (1xPBS containing 2% FBS and 0.1% sodium azide) at 4 °C for 30 min with PE-Cy7-conjugated CD45 antibody (clone 30-F11). Peripheral blood and bone marrow samples were also obtained from both groups of mice and analyzed for numbers of circulating leukocytes. Ab-stained cells were analyzed on LSRFortessa flow cytometer (BD Biosciences), and the data was analyzed with FlowJo 10.1r5 software. All the antibodies were obtained from BD Biosciences (San Diego, CA).

### Statistics

Statistical analyses were done using Prism software (GraphPad, La Jolla, CA). Analyses were done using unpaired Student *t* test with two-tailed *p* value. *p <* 0.05 was considered significant. Data are presented as mean ± SEM. For Western blot data, a representative blot is presented.

## Results

### miR-15a expression was decreased in high glucose conditions in REC

**Fig. 1:**
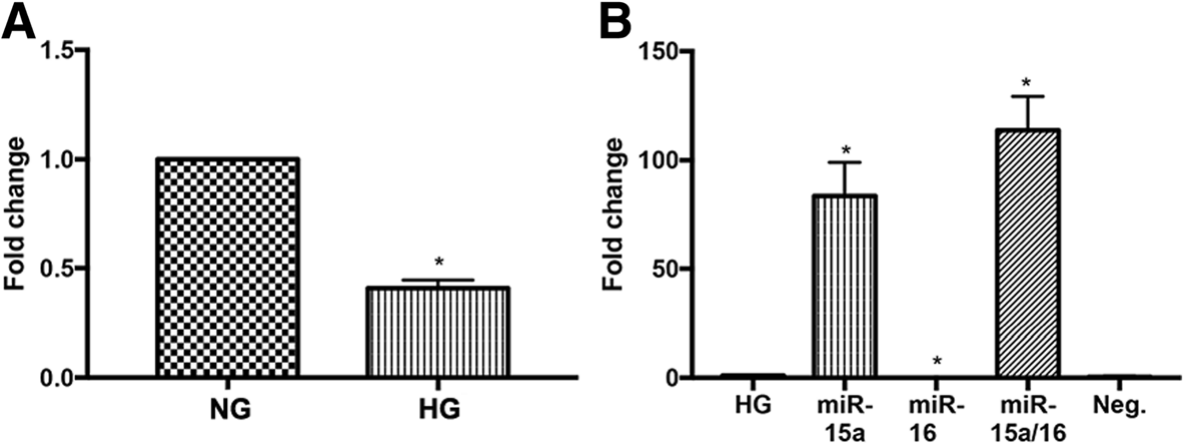
Decrease of miR-15a expression in high glucose conditions, and transfection-induced fold changes. **a** Fold change of miR-15a expression is shown. After 3 days of REC culture in high glucose (25 mM) medium, miR-15a expression was reduced (0.4-fold reduction) compared to that in normal glucose (NG; 5 mM) group. **b** Fold changes of miR-15a/16 after transfection in REC. REC were transfected with mimics (30 nM of final concentration) of miR-15a and/or miR-16 in high glucose conditions. Approximately 84- and 114-fold increases of miR-15a levels were detected following transfection with miR-15a and miR-15a/16 mimics, respectively, compared to HG control. *p < 0.05 versus HG, *N* = 3; data are mean ± SEM

Our previous study demonstrated a 0.2-fold decrease in miR-16 levels in REC cultured under high glucose conditions as compared to normal glucose [9]. In the present study, we found that high glucose also decreased the miR-15a expression by 0.4-fold compared to normal glucose (Fig. 1a).

Since we found that high glucose conditions significantly decreased miR-15a and miR-16 expression in REC, we wanted to elevate the miRNA expression through transfection with miRNA mimics. The levels of miR-15a expression were increased 84-fold in REC treated with mimics compared to cells transfected with control (Fig. 1b). We previously reported that transfection with miR-16a mimics increased miR-16a expression by 54-fold compared to REC in high glucose-treated with control [9].

### miR-15a/16 reduced the levels of IL-1β and TNFα in high glucose conditions

**Fig. 2:**
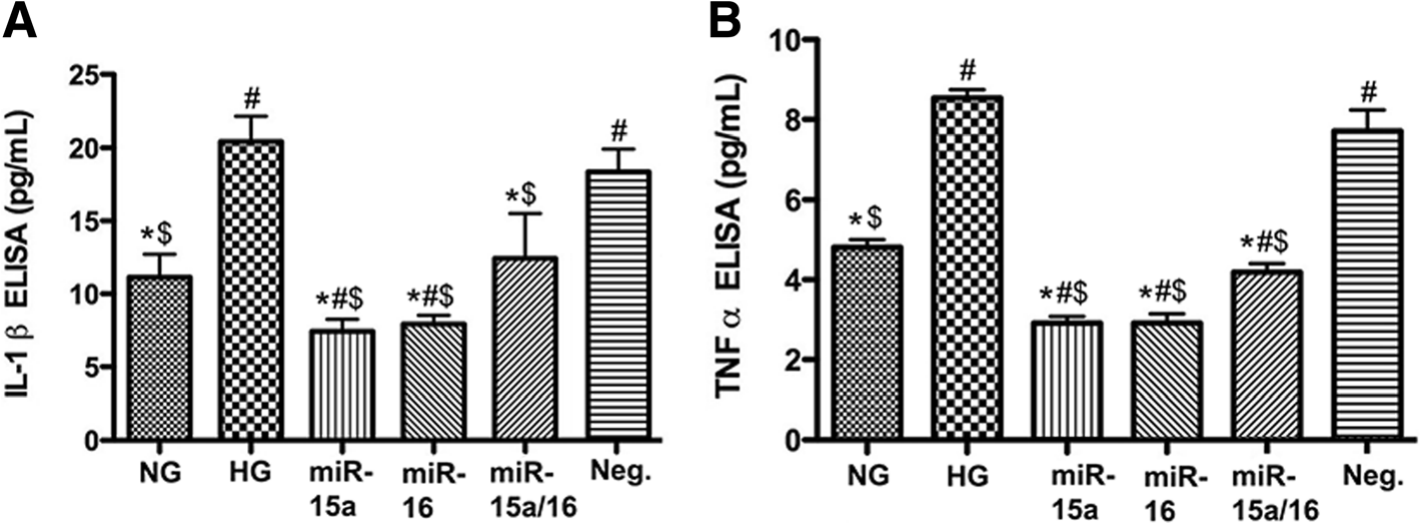
Assessment of IL-1β and TNFα levels in cultured REC. ELISA results for IL-1β (**a**) and TNFα (**b**) on REC in normal glucose (NG, 5 mM) or high glucose (HG, 25 mM) and transfected groups. miR-15a/16 reduced the levels of both IL-1β and TNFα significantly, compared to control HG condition. #*p* < 0.05 versus NG, *p < 0.05 versus HG, $*p* < 0.05 versus Neg., *N* = 4; data are mean ± SEM

Elevated levels of inflammatory molecules, including TNFα and IL-1β, have been shown in diabetic conditions [16, 21, 22]. Our previous study demonstrated that miR-15b/16 decreased TNFα levels in REC cultured in high glucose conditions [9]. In this study, we wanted to examine whether miR-15a/16 overexpression could decrease IL-1β and TNFα levels in REC cultured under high glucose conditions. We found that high glucose conditions increased both IL-1β and TNFα levels in REC. However, overexpression of miR-15a/16 resulted in significant decreases of the inflammatory signaling in REC (Fig. 2), suggesting miR-15a/16 plays a role in suppressing pro-inflammatory signaling, specifically IL-1β and TNFα, in REC under high glucose conditions.

### miR-15a/16 suppressed NF-κB activation in high glucose conditions

Our previous study has shown that the phosphorylation of NF-κB p65 (Ser 536) was increased in REC exposed to high glucose [12]. miR-15a is predicted to target TLR5/and 8, and the TLR signaling mediates the activation of NF-κB [18–20, 23, 24]. Additionally, TNFα is known to activate NF-κB [25, 26].

**Fig. 3:**
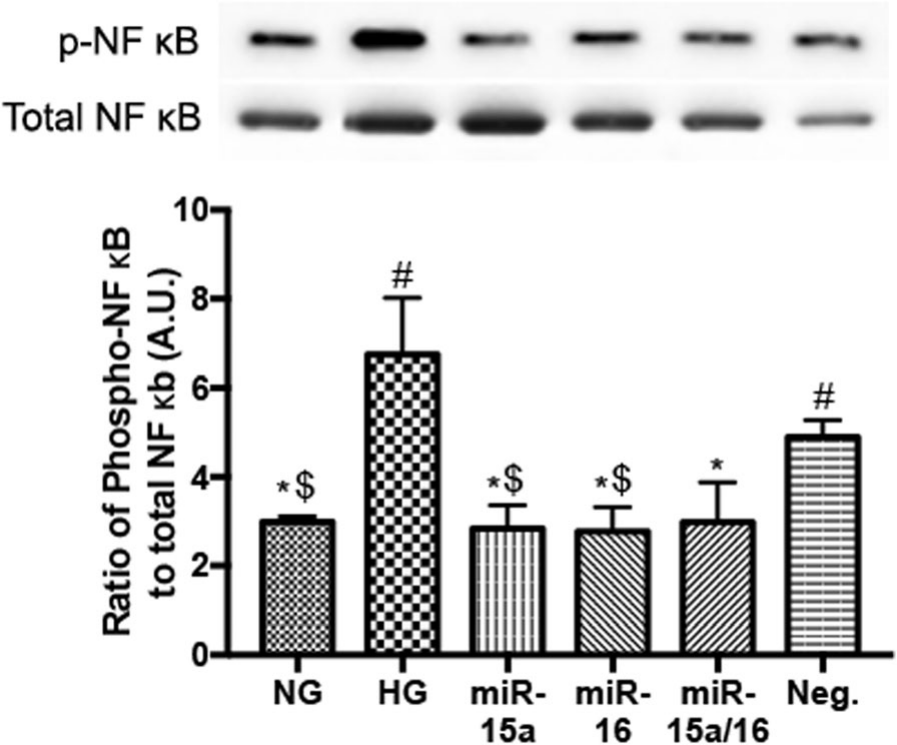
Effects of miR-15a/16 on NF-κB (Ser 536) phosphorylation in vitro. REC were cultured in normal glucose (5 mM, NG) or high glucose (25 mM, HG) and transfected groups. miR-15a/16 decreased the levels of NF-κB phosphorylation significantly, compared to control HG condition. #*p* < 0.05 versus NG, *p < 0.05 versus HG, $*p* < 0.05 versus Neg., *N* = 3; data are mean ± SEM. A representative blot is shown

Thus, we investigated whether NF-κB levels in REC were reduced in high glucose conditions after transfection with miR-15a/16 mimics. Our results demonstrated that REC overexpressing miR-15a/16 showed reduced levels of NF-κB phosphorylation in high glucose conditions (Fig. 3). Therefore, our in vitro study suggests that miR-15a/16 plays a role in the suppression of pro-inflammatory signaling in high glucose conditions.

### miR-15a/16 inhibited leukostasis in vivo

Our in vitro data suggest that miR-15a/16 play a role in inhibiting pro-inflammatory signaling in diabetic conditions. Investigating the role of miR-15a/16 in vivo is critical to confirm protective effects of the miR on the diabetic retina. To generate conditional knockout mice in which miR-15a/16 is eliminated in vascular endothelial cells, we crossed miR-15a/16 floxed mice with cdh5-Cre mice. After two generations, tissues from ear punch of 2-week-old mice were collected and processed for genotyping. PCR genotying showed DNA bands from three different groups of mice: wild-type, heterozygous, and homozygous mutant. Results demonstrated that mutant mice, miR-15a/16 Cre-LoxP, were positive for Cre and homozygous for floxed allele (Additional file 1).

**Fig. 4:**
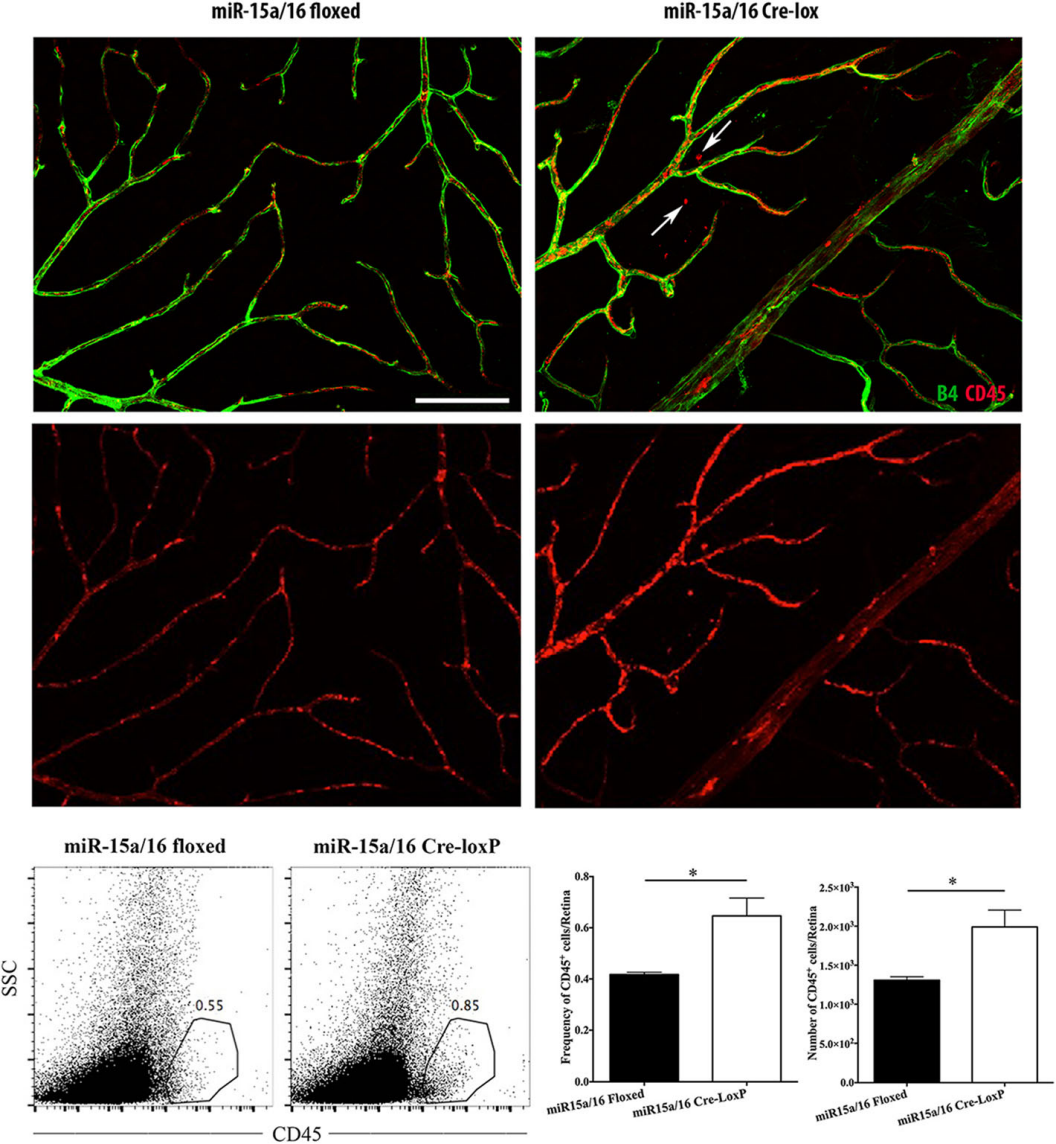
Effects of miR-15a/16 on retinal leukostasis. Mouse retinas were flat mounted and immunostained with CD45 (leukocytes, *red*) and isolectin B4 (blood vessels, *green*). miR-15a/16 Cre-LoxP mice showed intense leukostasis compared to the miR-15a/16 floxed mice. More CD45-positive cells were observed in miR-15a/16 Cre-LoxP mice than miR-15a/16 floxed mice, and some of CD45-positive cells were found outside of the vessels (*arrows*). *N* = 5 mice for each group. Representative FACS plots denoting the frequencies of CD45^+^ leukocytes in retinal tissue. Bar diagrams denote the frequencies and number of CD45^+^ leukocytes. Data were analyzed using two-tailed Student *t* test (*p < 0.05). Data are mean ± SEM. *N* = 4 (miR-15a/16 floxed), 3 (miR-15a/16 Cre-LoxP)

We studied whether miR-15a/16 plays a role in reducing leukostasis in the retina. Retinas were isolated from miR-15a/16 floxed and miR-15a/16 Cre-LoxP mice at 3 months of age. Immunostaining was performed on whole mount retina to label leukocytes and blood vessels using antibodies against CD45 and isolectin B4, respectively. We found larger numbers of CD45-labeled leukocytes in miR-15a/16 Cre-LoxP mice compared to miR-15a/16 floxed mice (Fig. 4, top panels). Therefore, our results demonstrated that miR-15a/16 play a role in reducing retinal leukostasis in vivo. To get a more quantitative view of whether miR-15a/16 regulate inflammatory cells, we performed flow cytometry on retinal tissue separated from unmanipulated eyes of miR-15a/16 Cre-LoxP and miR-15a/16 floxed mice. Retinal tissues underwent enzymatic digestion to obtain a single-cell suspension for flow cytometry. As is shown in Fig. 4 bottom panels, the loss of miR-15a/16 in the retina led to a significant increase in the frequency and absolute number of CD45+ leukocytes. Collectively, our results showed a moderate but statistically significant increase in the influx of CD45+ leukocytes in the retinal tissue lacking miR-15a/16 expression.

**Fig. 5:**
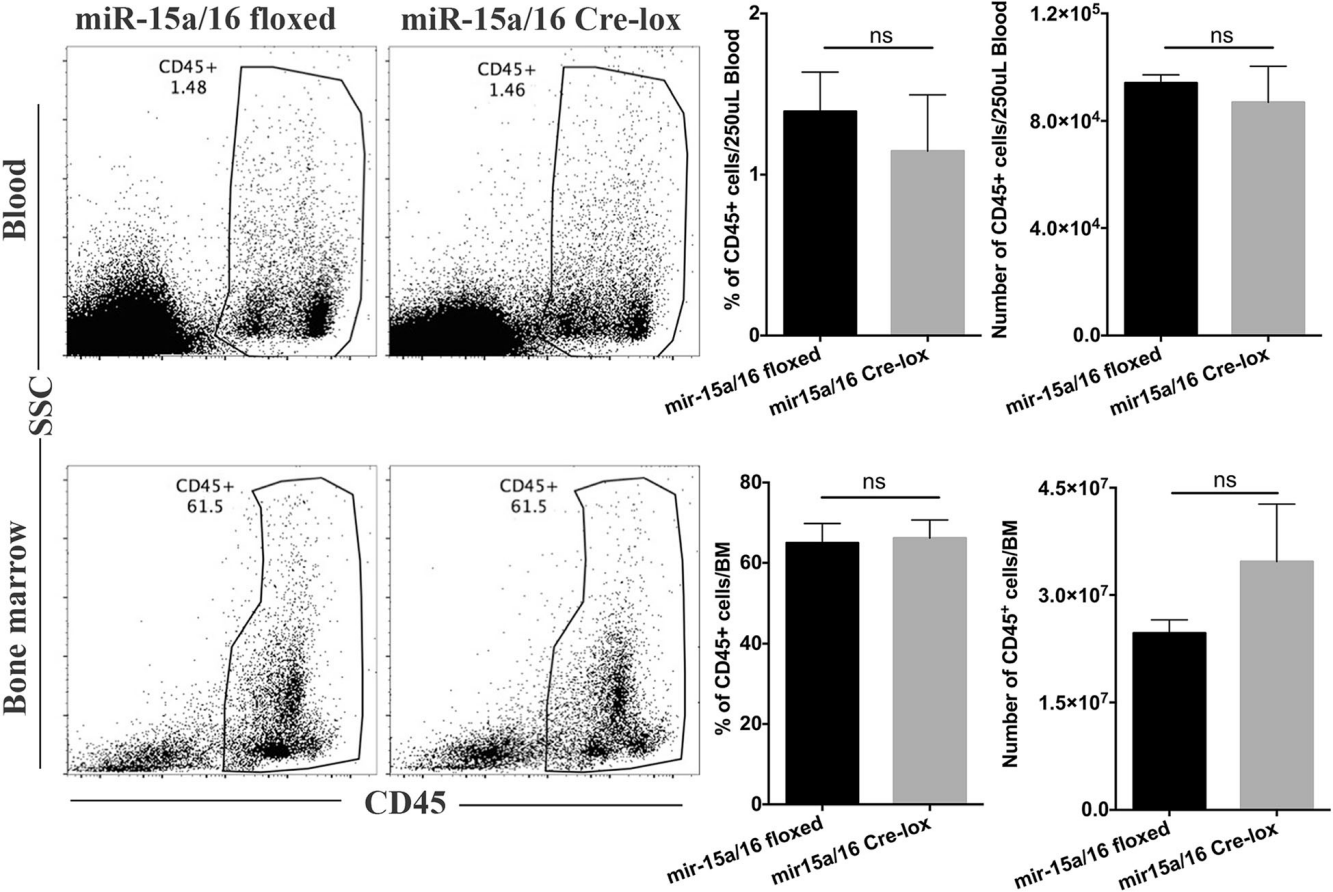
Effects of miR-15a/16 on the number of circulating leukocytes. Flow cytometry analysis was performed using the peripheral blood and bone marrow samples collected from control floxed and miR-15a/16 Cre-loxP mice. No significant difference was found in the frequency and absolute number of circulating leukocytes between the two groups of mice. Bar diagrams denote the frequencies and number of CD45^+^ leukocytes in peripheral blood and bone marrow. Data were analyzed using two-tailed Student *t* test (ns = non-significant). Data are mean ± SEM. *N* = 3 (miR-15a/16 floxed), 3 (miR-15a/16 Cre-LoxP)

Additionally, we determined whether miR-15a/16 Cre-loxP mice have an increased number of circulating leukocytes compared to control floxed mice. To assess this, flow cytometry was carried out on the peripheral blood and bone marrow samples obtained from control floxed and miR-15a/16 Cre-loxP mice. Our results showed no significant difference in frequency and absolute number of circulating leukocytes, when compared between both groups of mice (Fig. 5). These results clearly indicate that circulating pool of leukocytes is not accountable for an increased accumulation of CD45+ cells seen in the retinal tissue of miR-15a/16 Cre-loxP mice.

### miR-15a/16 suppressed IL-1β and TNFα signaling in vivo

**Fig. 6:**
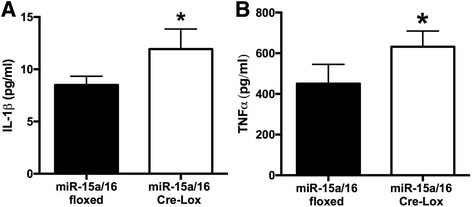
Changes of IL-1β and TNFα levels in the retina. ELISA was performed using retinal lysates. Increased levels of IL-1β (**a**) and TNFα (**b**) were found in miR-15a/16 Cre-LoxP mice compared to the miR-15a/16 floxed mice. *P < 0.05 vs floxed. *N* = 5 for all groups. Data are mean ± SEM

As we showed inhibitory roles of miR-15a/16 on pro-inflammatory signaling in cultured REC, we next investigated whether miR-15a/16 has the same effects on the levels of IL-1β and TNFα in vivo. At 3 months of age, we collected whole retinas from miR-15a/16 floxed and Cre-LoxP mice and analyzed the retinal lysates using ELISA. Our results demonstrated that the levels of IL-1β and TNFα were significantly increased in the miR-15a/16 Cre-LoxP mice compared to miR-15a/16 floxed mice (Fig. 6). Thus, data strongly suggest that miR-15a/16 play a role in the suppression of pro-inflammatory signaling in the retina.

### miR-15a/16 inhibits NF-κB activation in vivo

**Fig. 7:**
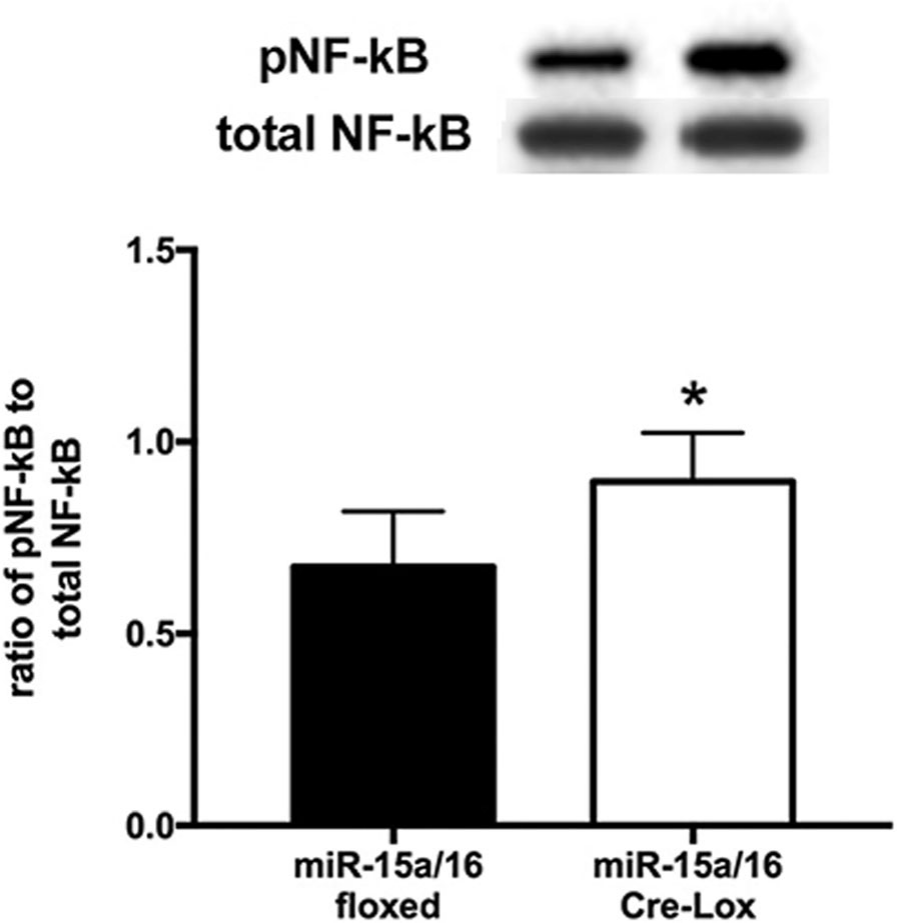
Effects of miR-15a/16 on NF-κB (Ser 536) phosphorylation in mouse retina. Higher levels of NF-κB were found in miR-15/16 knockout mice compared to miR-15a/16 floxed mice. *P < 0.05 vs. floxed. *N* = 5 for all groups. Data are mean ± SEM. A representative blot is shown

In addition to the inhibitory effects of miR-15a/16 on the levels of pro-inflammatory cytokines, we also wanted to confirm the regulatory roles of miR-15a/16 on the phosphorylation of NF-κB in vivo. NF-kB phosphorylation was significantly higher in the retina from miR-15a/16 Cre-LoxP mice compared to miR-15a/16 floxed mice (Fig. 7). The results indicate that loss of miR-15a/16 in retinal endothelial cells induces increased levels of NF-κB phosphorylation. Thus, the outcome suggests that miR-15a/16 play an important role in reducing retinal leukostasis, through suppressing inflammatory signaling of IL-1β, TNFα, and NF-κB in the retina.

## Discussion

Diabetic complications in the retina involve many factors, including activation of pro-inflammatory signaling, endothelial dysfunction and death, and apoptosis of retinal cells [2, 27, 28]. Yet we have an incomplete understanding of the molecular regulators that play a role in the pathological mechanisms of diabetic retinopathy. In this study, we focused on miRNA as a mediator of retinal inflammation, as an emerging regulator of signaling pathways involved in diabetic retinopathy.

Our previous studies and literature implicate the involvement of miR-15a/16 in the pathology of diabetes. In our studies, we found that REC cultured in high glucose conditions had decreased levels of miR-15a/16. This is consistent with previous studies showing the reduction of miR-15a levels in HUVEC cultured under high glucose conditions [8] and in the plasma of patients with prevalent DM [7]. However, eyes with PDR showed increased levels of hsa-miR-15a [10], which may reflect a key characteristic of miRNA, where expression and function are both cell- and tissue-specific.

Activation of pro-inflammatory signaling is a key factor that contributes to the pathological responses of diabetic retinopathy [3, 28]. miR-15a/16 are inhibitory to TLR4 inflammatory signaling in mouse macrophages treated with LPS [17]. Furthermore, we previously had shown that miR-15b/16 overexpression suppress TNFα signaling in REC in high glucose conditions [9]. In the present study, we showed that miR-15a/16 play a significant role in the reduction of pro-inflammatory signaling (IL-1β and TNFα) in vitro, as well as in vivo. TLR pathways mediate the activation of NF-κB [18, 23, 24]. NF-κB is another important signaling molecule that contributes to inflammation in diabetic retinopathy [29–31]. Our previous study demonstrated that TLR4 and NF-κB signaling were reduced in REC when miR-146a was overexpressed in high glucose conditions [12]. In the current study, we demonstrated that NF-κB activation was inhibited by miR-15a/16 overexpression in cultured REC with exposure to high glucose, which was confirmed in our miR-15a/16 conditional knockout mice.

Previous studies have shown that hyperglycemia and high glucose conditions induce leukocyte adhesion to endothelial cells [32, 33]. Work has shown that miR-146a can reduce NF-κB activation, leading to decrease of leukocyte adhesion to REC [30]. However, little else is reported on the role of microRNA in retinal leukostasis. From our in vivo study using miR-15a/16 Cre-loxP mice, we found that the loss of miR-15a/16 resulted in significant increase of leukostasis and inflammatory cells in the retina. Our flow cytometry data of the bone marrow and blood showed a comparable number of circulating CD45+ cells in both control floxed and miR-15a/16 Cre-loxP mice. Even though retinas were not perfused prior to the analysis, our results suggest that circulating pool of leukocytes is not accountable for an increased number of CD45+ cells seen in the retinal tissue of miR-15a/16 Cre-loxP mice. However, the increased number of CD45+ cells seen in the retinal tissue could be the combination of leukocytes adhered to retinal blood vessels and the ones that have come out of the blood vessels to the retinal tissue. Therefore, our outcomes indicate that miR-15a/16 in REC play a crucial role in the regulation of leukocyte adhesion in vivo. Therefore, this is the first evidence we are aware of that miR-15a/16 can regulate retinal leukostasis, potentially by suppressing pro-inflammatory signaling.

As one potential molecular mechanism of miR-15a/16 regulation of retinal inflammation, altered insulin signaling could be a candidate. Downstream molecules of the insulin signaling pathway are predicted targets of miR-15a and miR-16 (targetscan.org). Increases in leukostasis have been associated with insulin resistance [34], and our previous study showed that miR-15b/16 played a role in the inhibition of insulin resistance via reduced TNFa and SOCS3 signaling and increased IGFBP-3 levels, resulting in REC protection from hyperglycemia-induced apoptosis [9]. It is possible that miR-15a and miR-15b exert similar functions as they share the same seed sequence [35], and we will explore this further in future studies.

Although we have not examined the efficiency of deleting miR-15/16 cluster from the vascular endothelium of miR-15a/16 Cre-loxP mice, our results from in vivo studies strongly support successful deletion of the miR-cluster in the vascular endothelial cells. Wang et al. [36] demonstrated clear effects of miR-15a on maintaining retinal permeability in the retinas of Tie2-miR-15a transgenic mice.

## Conclusions

Overall, we showed that high glucose conditions decreased expression of miR-15a/16 in REC. In such conditions, overexpression of miR-15a/16 decreased pro-inflammatory mediators IL-1β, TNFα, and NF-κB in cultured REC. Moreover, we demonstrated the loss of miR-15a/16 in retinal endothelial cells increased inflammatory signaling of IL-1β, TNFα, NF-κB, and retinal leukostasis in the retina of conditional knockout mice. Thus, we suggest that miR-15a/16 is a potential target for the development of therapeutic strategies on diabetic retinopathy.

## Additional file

Additional file 1: Genotyping of representative samples from ten mice. Tissues were collected from tail at 2 weeks of age. (Top) PCR bands showing WT, HET, miR-15a/16 Cre-loxP genotypes. (Bottom) PCR bands showing Cre-positive. (TIF 1249 kb)
