# Supplementary figures and images for: miR-15a/16 reduces retinal leukostasis through decreased pro-inflammatory signaling
Source: J Neuroinflammation. 2016 Dec 8;13:305. doi: 10.1186/s12974-016-0771-8 (PMC5146897; doi:10.1186/s12974-016-0771-8)

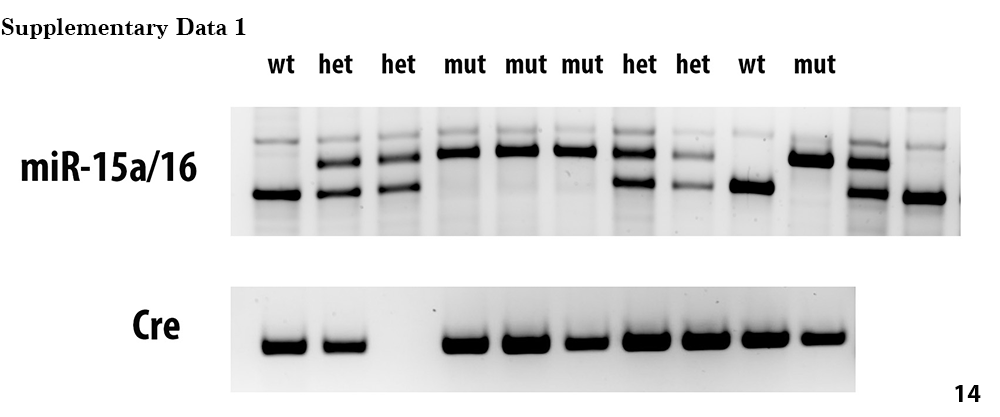

Supplement: Additional file 1: — Genotyping of representative samples from ten mice. Tissues were collected from tail at 2 weeks of age. (Top) PCR bands showing WT, HET, miR-15a/16 Cre-loxP genotypes. (Bottom) PCR bands showing Cre-positive. (TIF 1249 kb) [file 12974_2016_771_MOESM1_ESM.tif]
